# Assessing the Impact of Community Factors on Local Community Support for Tourism: An Empirical Investigation of the China-Pakistan-Economic Corridor

**DOI:** 10.3389/fpsyg.2022.893448

**Published:** 2022-04-29

**Authors:** Yunfeng Shang, Abdul Hameed Pitafi, Rao Muhammad Rashid

**Affiliations:** ^1^School of Hospitality Management, Zhejiang Yuexiu University, Shaoxing, Zhejiang, China; ^2^School of Management, Hefei University of Technology China, Hefei, China; ^3^Department of Management Studies, Bahria University, Karachi, Pakistan

**Keywords:** road and transport, CPEC, social media use, local residents’ community dimensions, tourism development

## Abstract

This research probes the influence of the construction of the China-Pakistan Economic Corridor (CPEC) on the tourism development behavior of local residents. By applying social exchange theory (SET), this study examines the impact of the community dimension on tourism development behavior through overall attitude. In addition, this study also examines the use of social media as a moderator in the relationship between overall attitude and tourism development. A survey tool has been used to obtain data from the people of Gilgit-Baltistan. Hypotheses were examined using the structural equation method (SEM) on 417 survey responses. The findings indicated community satisfaction and an overall positive attitude to the effects of development, although a negative attitude regarding concerns about community resources. Overall, attitude has a significant impact on tourism development behavior. Additionally, the use of social media strengthens the relationship between overall attitude and behavior that favors tourism development.

## Introduction

The construction of road and transport infrastructure has been recognized as an important driver of tourism development around the world (Kanwal et al., 2020b). It enhances the flow of tourists and increases business and economic opportunities for the local community. Research has found that the development of road and transport networks not only improves the attractiveness of current tourism sites but also directly affect the construction of new tourist sites in that area (Kanwal et al., 2020b). Previous studies have demonstrated a strong association between infrastructure development and the flow of visitors in an area (Khadaroo and Seetanah, 2007; Kurihara and Wu, 2016; Nazneen et al., 2019). For example, Khadaroo and Seetanah (2007) have noted that road and transport services have a major effect on tourism activity. Kurihara and Wu (2016) studied the relationship between tourism activities and high-speed rail and found that the high-speed rail services have greatly increased the movement of visitors to China and Japan. Specifically, better roads and transportation ease travel and facilities the tourist movement within the tourism location. On the other hand, poor roads and transport facilities at the destination, demoralize the visitors and they may not want to visit the location again (Nazneen et al., 2019).

With the development of the China-Pakistan Economic Corridor (CPEC), it is projected that Gilgit-Baltistan will be a center of tourism in Pakistan. Gilgit-Baltistan contains many natural landscapes, including lakes, glaciers, resorts, and the highest mountain range in the world. However, with poor destination road and transportation facilities, this region is not attractive for international tourists. It is predicted that CPEC road and transport will play a significant role in the promotion of tourism activities in the area (Nazneen et al., 2019). In addition, previous studies have examined the antecedents that influence locals’ perceptions of tourism’s impact, including community attachment (Gursoy et al., 2002; Moghavvemi et al., 2020), community development (Nunkoo and Ramkissoon, 2011), economic impact (Yoon et al., 2001), community satisfaction (Kanwal et al., 2020b), and its relationship with the local community; however, the impact of road and transport has received less attention. Therefore, based on the previous literature, this research investigates the relationship between the community dimension and residents’ tourism development attitude in the context of road and transport infrastructure development.

Furthermore, a positive overall attitude from the local community is required for the smooth implementation and execution of construction projects. Analysis has demonstrated that projects cannot succeed without the support and commitment of local people (Pappas, 2014; Sun et al., 2020). It is also necessary to project an optimistic image of the construction project to the local community. Scholars indicate that the use of print media or social media is beneficial in creating a positive image of construction projects among communities and individuals (Pitafi et al., 2018a,2020c; Allauddin et al., 2020). In addition, previous studies that have investigated the success factors of development projects—including economic benefits (Kanwal et al., 2019b) and business opportunities (Kanwal et al., 2019d; Nazneen et al., 2019)—have ignored the role of social media in constructing a positive image of projects in the mind of a local community. Social media may play a significant role in shaping a community’s overall positive attitude toward tourism development. Therefore, it is imperative to understand the role of social media in order to develop a deep understanding of resident’s pro-tourism development behavior.

The objective of this research is to examine the impact of the community dimension on pro-tourism development through overall attitude. It also explores social media’s moderating role in the relationship between overall attitudes and pro-tourism development, using data collect from the local residents of Gilgit-Baltistan in Pakistan. On the basis of SET, the behavior of the local population was analyzed in the light of road and transport developments. According to SET, local communities participate in and support infrastructure projects based on an exchange of resources. The outcomes of this study have several theoretical and practical implications. Theoretically, it contributes to the literature on tourism by investigating the tourism development behavior of local community members in the context of road and transport development. Secondly, we used social media as a moderator in the relationship between overall attitude and pro-tourism development, as research suggests that social media use may create a positive image for CPEC among the local community (Allauddin et al., 2020; Kanwal et al., 2020b). Thirdly, the findings here can help tourism development authorities as well as CPEC to plan future polices that keep local community perceptions in mind.

## Theoretical Framework

### Social Exchange Theory

The background of this research is the literature of social exchange theory (SET). According to SET, a person will respond positively to another entity if he/she has obtained benefits from other such instances (Ap, 1992). Several scholars have employed SET and investigated local residents’ reactions to tourism development (Perdue et al., 1990; Yoon et al., 2001; Kanwal et al., 2020a). Using SET, Kanwal et al. (2020a) identified a substantial relationship between benefits to the local community and local support in the context of CPEC. Perdue et al. (1995) also employed SET to examine local community support for tourism development. Similarly, Yoon et al. (2001) explored the benefits of tourism and support for further development and found a significant relationship. The key tenet of SET is that people will participate in an interaction if they believe that they are going to gain profit without incurring unacceptable cost. If they believe that the benefits are greater than the costs, they will likely be involved in the exchange and, therefore, promote more development in their communities.

Social exchange theory proposes that local people should participate in and promote the development of roads and infrastructure in their region. Research has shown that the construction of road and transport infrastructure has a significant impact relative to costs. Accordingly, this study suggests that host communities which see themselves as the beneficiary of tourism development are likely to view such development very favorably, whereas individuals who view themselves as suffering from losses or costs are likely to view tourism negatively.

## Literature Review

### Road and Transport Infrastructure

The construction of road and transport infrastructure has an important long-term effect on the development of a region. As a result, the development of road and transport infrastructure not only plays a key role in developing current tourist sites but can also establish other tourism-related activities (Kanwal et al., 2020b; Belova et al., 2022). In addition, previous studies have identified a substantial relationship between tourism activities and infrastructure development (Li et al., 2019; Nazneen et al., 2019). Access to tourist sites by road and transport services is critical for the development of tourism. Enhanced road and transport decreases travel time and costs that have a direct impact on the flow of visitors to destinations (Xu et al., 2022). On the other hand, if the chosen tourist site has poor roads and facilities, tourists can find alternative sites. This discussion suggests that road and transport are possible elements in the development of tourism and the promotion of tourism activities in an area.

In 2015, China initiated the CPEC project in Pakistan with expenditure of more than USD 60 billion (Kanwal et al., 2019d). CPEC is a mega-project that connects Gwadar in Pakistan with Kashgar in China by a road and rail networks. With the development of CPEC, it is believed that the Gilgit-Baltistan area will become a tourist center in Pakistan. Despite this area’s tourism potential, it is undeveloped and currently lacks adequate road and transport infrastructure. Therefore, the current research examines the impact of the community dimension on residents’ pro-tourism development behavior through their overall attitude.

### Resident-Community Dimensions

In recent decades, researchers have studied the interaction between individuals and their communities and have reported that geography and sociality are core elements of community. Previous research has also shown that traditions, living standards, and social customs are the main factors of community, and suggested that the participation of local people in community development can affect these relations (Trentelman, 2009; Zhang et al., 2020). Communities are groups of individuals with common interests, close interpersonal relationships, and shared reactions (Sharpley, 2014). Scholars of tourism have recognized that economic impacts may change the perception and attitude of community members toward tourism development (Zhang et al., 2020). Local communities are key stakeholders who actively determine the costs and benefits of tourism for their communities and themselves (Stylidis and Terzidou, 2014; Nunkoo and Gursoy, 2016). For example, Nunkoo and So (2016) observed that the personal benefits of tourism development could affect community members’ perceptions of its costs and benefits. On the other hand, McGehee and Andereck (2004) concluded that potential benefits do not substantially predict support for tourism development and that other factors influence community attitudes, including tradition and geographic location. This study, therefore, investigates the influence of the community dimension on overall attitudes toward road and transport development arising from CPEC.

China-Pakistan economic corridor construction provides several benefits to local communities, including infrastructure development, accessible transport, business opportunities, and tourism to the region (Kanwal et al., 2020a). On the other hand, an increase of tourists in the region may also increase crime, noise, traffic problems, and environmental issues (Nazneen et al., 2020; Ramkissoon, 2020). Furthermore, mega-infrastructure development requires local resources: Pappas (2014) argued that the development of such projects requires a substantial investment of human and physical resources from the local community. This study therefore examines the impact of the community dimension, including community satisfaction and residents’ concerns about development and resources within the context of CPEC.

### Tourism Development Behavior

Tourism development activity is being evaluated as the ultimate outcome predictor of this analysis. SET concludes that locals are likely to support the development of tourism as long as they believe that its potential cost is lower than the benefits. Most locals recognize tourism as an economic activity; the results of previous studies show that locals have a positive attitude toward tourism development (Yoon et al., 2001; Nazneen et al., 2020). Local support for the construction of road and transport infrastructure is important. However, is dependent on benefits: if locals believe that the cost of a construction project is greater than its value then they will reject it; otherwise they will support it. Local populations evaluate development according to costs and benefits. Six constructs can be used to examine the overall attitude of local communities to pro-tourism development behavior.

## Hypotheses Development

### Resident-Community Factors, Overall Attitude

Previous research has shown that locals may interpret tourism either positively or negatively, depending on how they perceive the impact of the utilization of recreation resources. Local community attitudes should be positive to ensure the smooth development of a mega-project. Research indicates that local communities are concerned with their physical resources, including historical and social/cultural resources. They may oppose tourism development because they assume that it will trigger problems for them (Pappas, 2014; Yolal et al., 2016). Past surveys have showed that local residents expect tourism development to have detrimental impacts (Nunkoo and Gursoy, 2016), such as traffic problems, environmental damage, the destruction of natural beauty, and security issues.

Local community resources are also needed for CPEC construction. CPEC roads and transport pass through a number of villages where the agricultural land of local community may be required. Controversy is sparked by the eviction of local people from their homes, land repossession, property destruction, and environmental effects caused by CPEC development. A recent study has shown that residents with stronger concerns about community resources may be more sensitive to the impacts of tourism development (Ramkissoon, 2020; Zhang et al., 2020). In the Gilgit-Baltistan Region, current property prices are low but road and transport improvements will put the cost of property outside the reach of many in the area—local inhabitants will not be able to afford land in their own area. The locals have concluded that the way in which resources are utilized and consumed for construction projects does not benefit them, so they may resist the development of tourism in their area. On the basis of SET, we can say that the local community of Gilgit-Baltistan opposes the development of tourism because of its utilization of their resources. Accordingly, this analysis proposes the following hypothesis:

**H1a:** Community resource concerns are negatively related to overall attitude.

“Community satisfaction” is the subjective evaluation and overall feeling of people about the general environmental and social status of their community (Zhang et al., 2020). It is a key element which affects overall attitude and motivates the local community toward a development project (Kakar and Khan, 2020). Uysal (2016) noted that a positive response from a local community regarding an infrastructure project is significant for satisfaction. Scholars consider community satisfaction as strong predictor of an overall positive attitude toward tourism development (Nunkoo and Ramkissoon, 2010; Kanwal et al., 2020b). Prior research has indicated that the development of roads and transportation can improve the lives of local people because infrastructure development significantly improves local economies (Chaudhry et al., 2017). Scholars have observed an improvement in the living standards of local communities with the development of tourism (Yoon et al., 2001; Ramkissoon, 2020), which in turn increases the satisfaction of the local community. Therefore, scholars advocate more research to understand the relationship between community satisfaction and its overall attitude, and on resulting pro-tourism development behavior.

The construction of roads and transport is likely to widely affect the host population. For example, local communities may receive economic, educational and business benefits from the construction of CPEC. Better facilities may also attract international tourists in the region. This flow of tourism activities will likely stimulate the economy of the area through the construction of hotels, restaurants, markets and local transportation (Kanwal et al., 2019a). Research has shown that perceived environmental, cultural, economic, and social impacts change people’s perceptions to contribute to and support tourism development under the framework of CPEC (Ali L. et al., 2018). Therefore, we expect that the benefits accruing to the local community from the construction of roads and transport is likely to change the overall attitude of locals. This aligns with SET, implying that, when locals perceive benefits from the construction of roads and infrastructure, their overall attitude becomes more positive. Hence, this study proposes the following hypothesis:

**H1b:** Community satisfaction is positively related to overall attitude.

Community development is another important parameter for assessing the overall attitude of the local community. The construction of road and transport infrastructure has a substantial impact on the local community’s living standard (Kanwal et al., 2020b) and tourism opportunities (Nazneen et al., 2020). Road and transport infrastructure construction is a leading antecedent to progressive community development which supports society, the economy, and the environment (Hadi et al., 2018; Ramkissoon, 2020). The literature argues that building infrastructure in developing countries promotes economic activity and attracts a flow of foreign investors, also benefiting the local community economically (Kanwal et al., 2019c). Thus, this study aims to explore the impact of CPEC infrastructure development on community development.

According to CPEC officials, construction in remote areas will also connect them with major cities, thus increasing urbanization (Ibrar et al., 2018, 2019; Kanwal et al., 2020b). Scholars propose that those living in or near a city have a higher living standards than others (Nunkoo et al., 2013; Hussain, 2018, 2020). CPEC can thus integrate and significantly contribute to the overall prosperity and development of an entire region (Idrees, 2015; Javaid, 2016). Roads and transport play a dynamic role in enhancing the economy of the country (Haq and Farooq, 2016). Numerous studies have investigated the impact of road and infrastructure development on educational institutions, health centers, and community development (Ali Y. et al., 2018; Ibrar et al., 2018), and reported that tourism development improves the overall infrastructure of a region. It is assumed that the development of road and transport networks would increase the tourism industry in the area, which will improve the lives of communities. By enhancing infrastructure, tourism development also provides opportunities for cultural interaction, more leisure amenities, and impacts the quality of life (Nunkoo et al., 2013; Nazneen et al., 2020). As local people benefit from the development of road and transport infrastructure, their overall attitude would become more optimistic. Based on SET, the overall positive attitude of the local population is linked to the benefits. Hence, this study suggests the following hypothesis:

**H1c:** Community development is positively related to overall attitude.

### Overall Attitude, Tourism Development Behavior

Earlier studies have found that the attitude of community residents correlates with actions or activities that can be both positive and negative (Carmichael, 2000; Balaguer and Cantavella-Jorda, 2002). Tourism research has examined the connection between the impact of tourism, overall community attitude, and support for further development, with mixed findings (Johnson et al., 1994; Carmichael, 2000; Yoon et al., 2001; Nunkoo and Ramkissoon, 2010; Peters et al., 2018). For example, Peters et al. (2018) indicated that the positive effect of tourism could affect the overall attitude of community members, providing greater support for development. Similarly, Yoon et al. (2001) have suggested that tourism’s benefits are positively linked to support for further development. Those who have benefited from development projects may have more positive attitude toward development (Nunkoo and Ramkissoon, 2010; Kanwal et al., 2019b).

In addition, research has found that the overall attitude of local populations could change with costs and benefits (Kanwal et al., 2019b; Nazneen et al., 2019). Jurowski et al. (1997) suggested that the overall positive attitude of residents is positively associated with support for further development. Similarly, Lee (2011) has proposed a significant relationship between benefits, opportunities, attitude and support, and reported a correlation between higher benefits, more positive attitudes, and greater support. Based on SET, residents’ overall attitude toward CPEC is based on its costs and benefits. The more positive the inhabitants’ attitude, the higher the pro-tourism development behavior. Therefore, the following hypothesis is proposed:

**H2:** Residents’ overall attitude is positively related to tourism development behavior.

### Moderating Role of Social Media Usage

Social media technology is an interactive forum where individuals and groups publish, upload, and modify user-generated content, and converse with others (Leonardi, 2014; Pitafi et al., 2018b). Being a web-based open forum, it can make a vital contribution to the credibility of the CPEC infrastructure project. Social media can help create a significant picture of society, products, and this construction initiative (Pitafi et al., 2020b; Wei et al., 2020), strengthening a positive image of tourism development in the mind of the local community. Therefore, we use social media as a moderator in the relationship between residents’ overall attitude and pro-tourism behavior. Due to the rapid development of the Chinese economy and the success of their infrastructure projects, CPEC is constantly discussed in national/international media reports; these discussions may continuously shape local residents’ overall attitude. While the level of awareness of ordinary people is unknown, social media can provide a picture of tourism development that can be evaluated (Kakar and Khan, 2020). This research has especially considered some aspects of politics where the image of CPEC is less positive and poses some important concerns in the minds of local residents. The persistence and increased use of social media may change the overall attitude of the local community, cultivating more support for tourism development.

Furthermore, the use of social media technologies allows individuals to express their valuable feelings, share comments, and even exchange details of tourism infrastructure development (Leonardi, 2015). In particular, social media usage plays an important role in raising awareness among local communities about tourism development (Wong et al., 2016; Pitafi et al., 2018a). Local communities are a major stakeholder in the success of tourism development, so it is essential that they develop a positive perception of tourism (Saad et al., 2020). Lack of coordination and collaboration will lead to further delays and challenges in the implementation of the CPEC development project while the use of social media can enhance its overall support within local communities (Surahio et al., 2022). In addition, if a positive perception of tourism is generated among Pakistani locals through social media, their overall attitude toward tourism development will be more positive. Therefore, this analysis proposes the following hypothesis:

**H3:** Social media use positively moderates the relationship between overall attitude and tourism development behavior such that, the higher the use of social media, the stronger the relationship between overall attitude and **tourism development behavior**.

## Research Methodology

### Study Location

**FIGURE 1:**
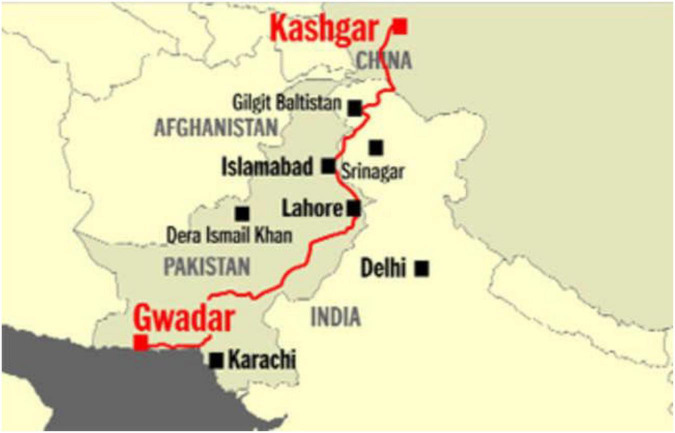
Location of Gilgit-Baltistan.

The focus of this research is the citizens of Gilgit-Baltistan, the tourism center and gateway of China, and Pakistan. Gilgit-Baltistan, recognized as Pakistan’s tourism hub, consists of a range of mountains, occupying 72,496 square kilometers, and connecting Pakistan with China, Afghanistan, and India-controlled Kashmir. This area has a fascinating history and is one of the world’s most distinctive geographic regions. It is mostly mountainous, with an amazingly beautiful landscape and visitor attractions. Gilgit-Baltistan is one of Pakistan’s most populous areas, with numerous tourist attraction sites (Nazneen et al., 2019). Its natural scenery and geographic wonders encourage both domestic and international visitors. The area of Gilgit-Baltistan consists of many beautiful sceneries, including the highest mountain range of the world (Himalaya, Karakoram Hindu kash), the second highest K2 point, and the longest glaciers. However, inadequate infrastructure facilities in the area tourism is not promoted well. Its assumed that the construction of CPEC may improve the infrastructure services, beauty of region in the region and may enhance the tourism activities in near future. Figure 1 depicts a map of Gilgit-Baltistan.

### Data Collection Procedures

In order to fully realize the objective of this research, the authors have adopted a quantitative approach and obtained data by utilizing a survey questionnaire (Hinkin, 1998). A survey method also aids an understanding of the behavior of constructs. The focus community of this research is the citizens of Gilgit-Baltistan, the CPEC tourism center and gateway. The author used English to prepare the questionnaire because the academic language of Pakistan is English. We aided participants if they had difficulty in interpreting the questions. The survey was divided into two sections. The first consisted of demographic information, along with a cover letter which indicated the overall purpose of the research and the concepts of the measurement items. The second section comprised the items of all the constructions. This research chose educated participants because illiterate people may not have enough knowledge of CPEC. It was a convenient sampling process since the educated population is easily reached by this method. The authors verified the consistency and clarity of the questionnaire in a number of ways. The complete questionnaire was firstly verified by a professor of English for errors in language. The contents of the survey items were then examined by five professors from the School of Economics and Management. Following their suggestions, the authors significantly modified the content of the questionnaire. Finally, ethical committee of university also has approved the objective of this study. In addition, authors has followed the ethical guidelines of Zhejiang Yuexiu University; Shaoxing, Zhejiang China and Hefei University of Technology China. This study has approved by the ethical committee of Zhejiang Yuexiu University; Shaoxing, Zhejiang China and Hefei University of Technology China. The ethical committee also has waved the consent of this study. A pilot study was performed on 51 respondents: the findings were considered satisfactory, with a Cronbach’s alpha higher than 0.700 for each construct, which encouraged us to proceed.

From August to November 2019, 600 hard-copy questionnaires were distributed across Gilgit-Baltistan. The authors visited educational institutions (schools/colleges/universities), government offices, industry firms, restaurants, and CPEC construction sites for data collection purposes. Over these four months, 500 questionnaires were received with a response rate of 83.33%. 83 responses were omitted from the final dataset because they were incorrectly completed or because several of the entries were blank; the remaining 417 relevant responses were used for the final study. The overall information from the participants is in Table 1. The participants comprised 270 males (64.70%) and 147 females (35.30%). The age range of the respondents was over 20 years of age, since those below 20 may not be able to accurately forecast the benefits of CPEC infrastructure.

**TABLE 1:** Demographic information of the samples.

Variables	N	Percentage	Variables	N	Percentage
**Gender**			**Occupation**		
Male	270	64.70	Government employee	88	21.10
Female	147	35.30	Private employee	96	23.00
**Age**			Business people	67	16.10
21–30 years old	130	31.20	Student	41	9.80
31–40 years old	125	30.00	House wife	48	11.50
41-50 years old	102	24.50	Others	77	18.50
> 50 years old	60	14.40	**Income**		
**Education Level**			10000–25000 rupees	120	28.77
College degree or below	141	33.80	26000–50000 rupees	134	32.10
Bachelor’s degree	175	42.90	51000–75000 rupees	100	24.00
Master’s degree or higher	97	23.30	76000 to 100000 rupees	33	7.90
			>100000	30	7.20

### Research Instruments

To validate and realize the objective of the study, the author used several scales and instruments. The survey utilized a five-point Likert scale, ranging from “Strongly agree” to “Strongly disagree”. We used a five-point rather than a seven-point Likert scale because previous research suggests that the former is easier for respondents (Pitafi et al., 2020c; Wu et al., 2020). The research model of this study consists of eleven constructs, including control variables. The constructs used in the research model are pro-tourism development behavior, overall attitude, social media usage, community resource concerns, community development, and community satisfaction. The instruments used in this study were adopted from previous studies, although the contents of some items were slightly modified in the context of the CPEC project. The details of all the measurement items are shown in Appendix A.

#### Tourism Development Behavior

This dependent variable was measured using four items from Nazneen et al. (2020). The tourism development behavior construct measures residents’ overall attitude related to CPEC infrastructure development. The sample items is “I am willing to promote my region as a tourist destination with the development of CPEC road transport.”

#### Social Media Usage

This construct was used as moderator variable in the research model. The scale of social media usage consists of three items and was adopted from Kakar and Khan (2020). This construct measures the use of social media technology by residents for sharing and obtaining information related to the CPEC development. The sample items is “I often use social media to obtain CPEC-related information and knowledge.”

#### Overall Attitude

This consisted of three items and was borrowed from Peters et al. (2018). The scale measured the overall attitude of locals to CPEC development. The sample item is “In general, the advantages resulting from CPEC development outweigh the disadvantages for local residents in the region.”

#### Community Resource Concerns

The scale of these concerns was measured using six items from Delamere et al. (2001), and Sun et al. (2020). They measured the community’s overall local resource concerns used in CPEC construction. The sample item of this construct is “The CPEC development is a source of negative competition between my community and neighboring communities.”

#### Community Development

This measurement consisted of eight items adopted from Andereck and Vogt (2000) which measured the development of regions with the CPEC project. The sample item of this construct is “I support CPEC as having a vital role in this community.”

#### Community Satisfaction

This was measured using four items from Kanwal et al. (2020b) which measured the overall satisfaction of the local community related to the CPEC project in the region. The sample item of this scale is “I am pleased with the economic development that CPEC provides to our region.”

#### Control Variables

To measure the actual impact of independent variables on dependent variables, we used the gender, age, education, occupation and income of respondents as control variables (Pitafi et al., 2020a).

## Results and Analysis

To analyze the data, the authors initially screened 417 responses on SPSS 21.0 and did not find any missing or any outliers in samples. Hence, the data was suitable for further analysis. The analysis was conducted in three stages. Firstly, the author analyzed the validity, reliability, and factor loading of all the constructs. In the second stage, the author applied structural equation modeling to analyze the relationship among constructs. Finally, the PROCESS macro tool was used for moderation analysis.

### Validity and Reliability

The research model of the study was statistically validated using SPSS and AMOS software as guided by previous studies (Fornell and Larcker, 1981; Hair et al., 2019; Pitafi et al., 2020b). SPSS is an advanced statistical software tool, and use to analyze data in order to address complicated business and research challenges in a user-friendly environment (Pitafi and Ren, 2021). In this study, the descriptive statistics were computed using SPSS software. In addition, scholars also suggests AMOS software for obtaining confirmatory factor analysis (CFA) and SEM (Nachtigall et al., 2003). AMOS also has a graphical user interface that allows users to easily compute path diagrams, estimate parameters, and compute model fit values (Byrne, 2013). The reliability of the research model was analyzed using Cronbach’s alpha (CA), composite reliability (CR), and average variance extracted (AVE). CA is acceptable when it has a value higher than 0.700 (Fornell and Larcker, 1981). The results in Table 2 confirm that all constructs have CA values ranging from (0.742–0.891), thus higher than 0.700. CR is appropriate if the value is greater than 0.700. Table 2 shows that all constructs have CR values of (0.816–0.916), higher than 0.700. Similarly, AVE is adequate with values higher than 0.500; the table indicates AVE scores which range from (0.566–0.661) (Hinkin, 1998). Additionally, the convergent validity model was evaluated using standard factor loading. The literature recommends that standard factor loading of all constructs should be higher than 0.600 for the acceptable convergent validity of the research model. Table 2 indicates that all items of all constructs have a loading higher than the minimum threshold value of 0.600, ranging from (0.621–0.946) (Fornell and Larcker, 1981). Hence, the proposed conceptual model possesses an acceptable level of reliability and convergent validity.

**TABLE 2:** Results of confirmatory factor analysis.

Construct	Items	Loading	CA	CR	AVE	MSV	ASV
Tourism development behavior	4	0.621	**0.819**	**0.883**	**0.661**	**0.062**	**0.0442**
		0.946					
		0.684					
		0.946					
Social media usage	3	0.809	**0.787**	**0.853**	**0.659**	**0.266**	**0.094**
		0.790					
		0.836					
Overall attitude	3	0.678	**0.742**	**0.816**	**0.599**	**0.128**	**0.079**
		0.752					
		0.878					
Community resource concerns	4	0.780	**0.777**	**0.838**	**0.566**	**0.266**	**0.088**
		0.733					
		0.828					
		0.659					
Community development	8	0.763	**0.891**	**0.916**	**0.580**	**0.033**	**0.013**
		0.614					
		0.654					
		0.811					
		0.856					
		0.810					
		0.792					
		0.763					
Community satisfaction	4	0.900	**0.831**	**0.885**	**0.659**	**0.075**	**0.031**
		0.840					
		0.722					
		0.773					

*CA, Cronbach’s alpha; CR, composite reliability; AVE, average variance extracted; MSV, maximum shared variance; ASV, average shared variance; discriminant validity: AVE < MSV.*

The author tested the discriminant validity of the study model using the findings in Tables 2, 3. Table 2 shows that all constructs had MSV values higher than ASV, suggesting an acceptable degree of discriminant validity. Table 3 indicates that all AVE square-root values are higher than its inter co-relation, showing good discriminant validity for the research model (Fornell and Larcker, 1981).

**TABLE 3:** Means, standard deviation, and correlations.

Variable	M	SD	1	2	3	4	5	6	7	8	9	10	11
(1). Tourism development behavior	3.984	0.749	**0.813**										
(2). Social media usage	4.703	1.223	0.229	**0.811**									
(3). Overall attitude	3.471	0.826	0.248	0.358	**0.773**								
(4). Community resource concerns	2.616	1.013	−0.237	−0.516	−0.313	**0.752**							
(5). Community development	3.919	0.810	0.147	0.046	0.182	−0.010	**0.761**						
(6). Community satisfaction	3.576	0.796	0.161	0.152	0.275	−0.147	−0.080	**0.811**					
(7). Income	**NA**	**NA**	−0.003	−0.089	−0.148	0.124	0.028	−0.015	**NA**				
(8). Occupation	**NA**	**NA**	−0.107	0.081	0.045	−0.046	0.054	0.024	−0.463	**NA**			
(9). Education	**NA**	**NA**	−0.037	−0.043	0.081	0.098	−0.085	0.036	0.001	−0.082	**NA**		
(10). Age	**NA**	**NA**	0.011	0.126	−0.021	−0.104	0.065	0.101	−0.303	0.248	−0.432	**NA**	
(11). Gender	**NA**	**NA**	−0.072	0.141	0.130	−0.165	−0.121	0.048	−0.285	0.246	−0.0164	0.186	**NA**

*M, Mean; SD, standard deviation. Diagonal elements are AVE square root.*

Along with validity and reliability, we also analyzed the common method bias (CMB) problem using several tests. The literature recommends several strategies to solve potential CMB (Pavlou and El Sawy, 2006; Podsakoff et al., 2012). Therefore, the author first utilized some technological remedies, including the confidentiality of the responses, to resolve the potential problem of CMB. For example, scholars have suggested that the anonymity of responses could help to solve the potential problem of CMB. The cover letter with the surveys did state that the participants’ responses would remain confidential and that their feedback would only be used for research purposes. Secondly, the author applied some statistical methods to address the likelihood of CMB issue in the dataset. For example, Liang et al. (2007) suggested a procedure for analysis of CMB problems. Following this procedure, the results of the analysis indicated that the substantive factor had a score of 65.5% of the variance, whereas the method factor had a score of 1.3% of the variance, which indicates no possibility of a CMB problem in the current study. In addition, the results of Table 3 revealed that all variables had co-relation values of less than 0,600 (Pavlou and El Sawy, 2006), suggesting that CMB did not occur in the sample. Finally, the author also applied the variance inflation factor (VIF) test; the results revealed that VIF values were below the prescribed value of 3.3 (Pitafi et al., 2020c). This suggests that CMB does not pose a serious problem to the data set. Together, all the facts indicated that there was no CMB issue in the current data.

### Structural Model

**FIGURE 2:**
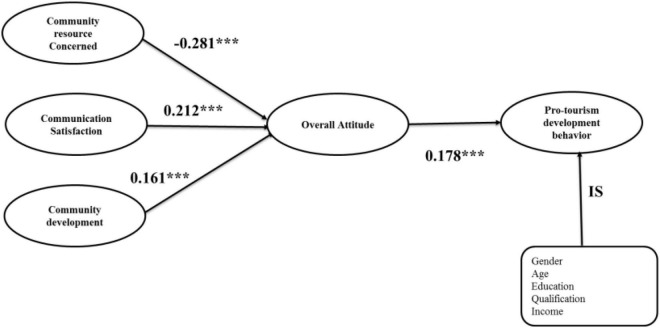
Structural model. ***p < 0.001, IS = Insignificant.

The theoretical research model of the present study was evaluated using AMOS 21.0 software (Hair et al., 2017), using a structural equation procedure with maximum likelihood. The findings of the structural model ensured that the model fit values are (CFI = 0.916, TLI = 0.901,IFI = 0.916, NFI = 0.875, AGFI = 0.850, REMSA = 0.06, CMIN/DF = 718.19/257 = 2.79), all within the recommended range (Hair et al., 2017). In addition, the effects of the structural equation modeling are represented in Figure 2 and suggest that all proposed hypotheses are confirmed in the present analysis. The findings show that community resource concerns a have negative effect on overall attitude (B = −0.280, *t* = −5.44, *p* < 0.001), while community satisfaction (B = 0.212, *t* = 4.817, *p* < 0.001) and community development (B = 0.161, *t* = 3.300, *p* < 0.001) have a positive effect on overall attitude; these, respectively, support H1a, H1b, and H1c. Similarly, overall attitude has significant effect on tourism development behavior (B = 0.178, *t* = 4.347, *p* < 0.001), so H2 is validated. Therefore, H1a, H1b, H1c, and H2 are supported by current data since all observations imply that beta values are in the suggested range, *t* > 1.64, and *p* values are less than 0.05. Furthermore, the findings indicate that all the control variables were insignificant.

### Moderation Analysis

PROCESS MACRO Model 1 was used to explore the moderating effect of social media use, as suggested by Hayes (2007). The findings in Table 4 show that social media use significantly strengthens the relationship between overall attitude and tourism development behavior with the interaction term (B= 0.168, *t* = 6.584, *p* < 0.001); thus, H3 is validated.

**TABLE 4:** Moderation analysis.

	B	SE	T	*R* ^2^
**Outcome:** Tourism development behavior				0.177
Constant:	4.436***	0.305	9.610	
Overall attitude	0.185****	0.033	4.028	
Social media use	0.136***	0.033	4.028	
Overall attitude * Social media use	0.168***	0.025	6.584	
Income	−0.031	0.034	−0.098	
Occupation	0.056	0.022	−0.2500	
Education	−0.087	0.052	−2.655	
Age	0.015	0.038	0.406	
Gender	−0.161	0.073	−2.214	

***p < 0.001.

**FIGURE 3:**
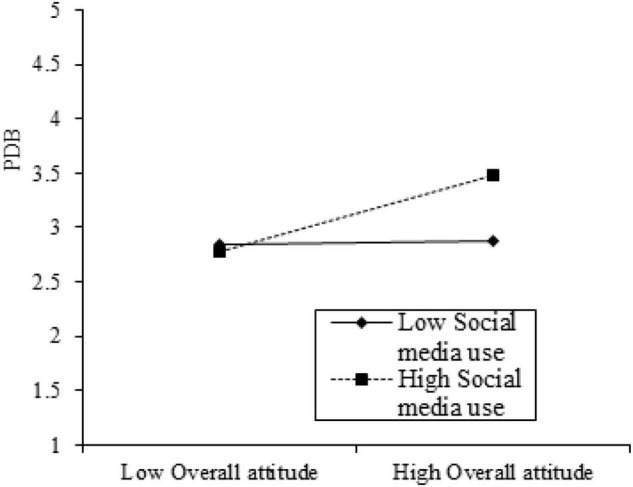
Moderating effect of social media use in the relationship between overall attitude and tourism development behavior. PDB = tourism development behavior.

In addition, a graphical approach was applied (Aiken et al., 1991) to understand the moderating role of social media use. Figure 3 indicates that social media use heightens the significant relationship between overall attitude and tourism development behavior. As verified in Figure 3, the moderating influence of social media usage on the link between overall attitude and tourism development was displayed with standard deviation (+ 1SD/−1SD) to indicate the influence of a high versus low degree of each.

## Discussion, Implications, Limitations

### Discussion

The objective of the current research is to assess the factors that influence residents’ tourism development behavior in Gilgit-Baltistan, Pakistan. By applying SET, we have examined the impact of the community dimension on tourism development behavior through local residents’ overall attitude in the context of road and transport construction. CPEC, under construction in Pakistan, connects Gwadar in Baluchistan, Pakistan to Kashgar, China through the Gilgit-Baltistan region in Pakistan. This region is a gateway of CPEC and full of natural scenery (Nazneen et al., 2020). However, due to poor road and transportation infrastructure and a lack of hospitality facilities, tourism activities in this area are limited (Kanwal et al., 2020a). Hence, it is expected that, with the construction of road and transport infrastructure under the CPEC framework, Gilgit Baltistan will become a tourism hub in the near future. The current dataset supports the proposed hypotheses of this study, suggesting that locals in Gilgit Baltistan have a positive attitude toward tourism because the local community’s income is dependent on tourism activities. Specifically, the findings indicate that community resource concerns have a negative impact on the local community’s overall attitude, supporting H1a. Previous studies indicate that locals are more concerned about their resources, culture, historical places, and living standards (Sun et al., 2020). The CPEC project is in its development stage and requires more resources from the local community; this may have a negative impact on locals’ overall attitude. Previous studies have also suggested similar results (Pappas, 2014; Kanwal et al., 2019b); for example, Nazneen et al. (2020) found that the perceived cost of tourism development has a negative impact on overall support for further development. In Hypothesis 1b, we suggested that local community satisfaction has a significant effect on overall attitude. The conclusions of this hypothesis are confirmed by the present research, which also relates to previous studies (Allauddin et al., 2020; Kanwal et al., 2020a). Accordingly, Kanwal et al. (2020b) observed that the personal benefit of road and transport projects had a significant impact on satisfaction, which subsequently enhanced the positive attitude of the local population. Results also indicated that community development with the construction of road and transport has a significant impact on overall attitude; H1c is also validated. Prior studies have also suggested similar results (Saad et al., 2020; Zhang et al., 2020). In addition, the study’s results also indicated that overall attitude has a significant impact on pro-tourism development behavior, so H2 is validated by the current dataset. Overall, the findings of this study reveal that SET is a reasonable theory for examining the pro-tourism development behavior of local communities.

Furthermore, this study also suggested a moderating role for social media in the relationship between overall attitude and tourism development behavior. Its results indicate that social media use reinforces the relationship between overall attitude and resident’s tourism development behavior, thus validating H3. Past studies indicate that social media use may convey a positive image of CPEC in Pakistan (Allauddin et al., 2020). The publicity on social media related to tourism development from CPEC is beneficial for constructing a positive picture of tourism development. For example, social media can highlight several benefits of tourism development, including infrastructure development, business opportunities, community development, health centers, and economic opportunities. These help eliminate the negative attitudes of the local community toward tourism development.

### Implications

By applying SET, this research examined the role of the community dimension in the development of tourism—not studied in the past, especially relating to road and transport development. The findings of this study have many implications, which may be useful for the development of tourism in Gilgit-Baltistan, Pakistan. In its theoretical implications, based on SET, the current study’s findings indicate that local community support for tourism development is based on its benefits, including community development (Ali et al., 2016; Kanwal et al., 2020a). This study’s results also assist our understanding of the local community’s perception of tourism development in the context of road and transport infrastructure. Additionally, by investigating the link between the community dimension and the overall attitude of local community, this study explores how road and transport construction is associated with tourism development in the region.

This study also has several practical implications. Firstly, its results indicate that community resource concerns have a negative impact on overall attitude. It is well-known that the construction of mega-projects requires local resources which may be perceived negatively by the local community (Pappas, 2014). For example, road and transport construction can destroy natural scenery, rivers, and historical sites, and may have a negative impact on local traditions (Kanwal et al., 2020b; Sun et al., 2020); therefore, alternative routes should be constructed to avoid damaging local property. Hence, CPEC officials should involve the local community in the construction phase, allocate some specific employment quotas, establish some tourism-specific development institutes, and also provide loan facilities to locals that can enable them to start their own tourism business. For example, a local community may establish some restaurants, travel agencies, and local transportation that helps to construct a positive image of CPEC.

Secondly, the results indicate that community satisfaction and development have a significant impact on the local community’s overall attitude. These findings confirm that the local community can understand that road and transport construction can benefits them in many ways. For example, it improves the scenic value, business and education in the region (Nazneen et al., 2019). Additionally, locals assume that the construction of roads and transport enhances the flow of tourists and increases economic opportunities, further leading to an overall positive attitude.

Thirdly, the study’s results indicate that social media use strengthens the relationship between overall attitude and tourism development behavior. These findings indicate that CPEC officials should effectively use social media technology to promote the post-tourism-related benefits for the local community. Research indicates that social media technology has the potential to construct positive as well as negative perceptions related to community development projects (Pitafi et al., 2020c; Rasheed et al., 2020). Frequent use of social media by CPEC can create positive awareness among the local population.

Finally, in order to foster tourism development support from the local community, it is recommended that CPEC officials organize tourism activities in conjunction with China. The Chinese government should also arrange some tourism trips for the locals of Gilgit-Baltistan, which may also help ameliorate the negative attitude of the local community. The government of Pakistan should organize some tourism-related conferences and also invite some international researchers to highlight the benefits of tourism development. Governments can encourage the development of tourism by advertising in the print media as well as on television—even arrange some CPEC-related talk shows on television, which may further help to publicize the development of tourism in Gilgit Baltistan.

### Limitations

Although the current findings have several implications, there are some shortcomings that must be addressed in future studies. Firstly, we conducted this study in Gilgit Baltistan, which is a tourism hub in Pakistan; thus, the generalizability of the findings is limited. The CPEC route is long and several other infrastructure projects are under construction in other parts of Pakistan. In addition, the respondents to the current study are highly educated participants who have more information on CPEC (Saad et al., 2020). Future studies should conduct research across Pakistan, with a large sample size and involve different communities.

Secondly, we investigated a few community dimensions in this study and did not examine the mediating effect. We also investigated social media’s role as a moderator in the relationship between overall attitude and tourism development and found a significant relationship. Future studies could examine other community dimensions, including community involvement, community attachment, community commitment and the social/cultural benefits to the community (Moghavvemi et al., 2020; Zhang et al., 2020). Researchers could also use a moderator which is related to tourism development, such as government support and economic benefits.

Thirdly, China benefits equally from the construction of CPEC, and China’s Xinjiang province is also a backward region in comparison to the rest of China (Kanwal et al., 2020a). Xinjiang also has several tourism sites which will benefit from the construction of CPEC. Hence, studies can involve Chinese communities and comparisons can be made.

Finally, we collected the data from the local population; it is recommended that future scholars include tourists as participants and investigate their perception of the CPEC construction project.

## Conclusion

The study has investigated the impact of CPEC, and especially the impact of CPEC development on tourism in a northern area of Pakistan (Gilgit-Baltistan). In the early stage of tourism development, residents may tolerate the costs of tourism (concerns about community resources) to obtain benefits from tourism development. Existing tourism studies related to CPEC focuses on economic sustainability but do not focus on the community dimension. The findings of this study indicate that perceptions of each of the three dimensions of community impact have distinct effects on overall attitude. Drawing on SET, this study concluded that the development of CPEC road and transport networks has a direct impact on the overall attitude of the local population. The findings of this study have validated all the proposed hypotheses, demonstrating that residents have an overall positive attitude toward tourism development. Its results indicate that local community development and satisfaction regarding infrastructure development have a significant impact on overall attitude. However, community resource concerns indicate a negative impact on the local community’s overall attitude. Overall attitude also has a positive impact on pro-tourism development behavior. In addition, social media use strengthens the relationship between overall attitude and resident’s tourism development behavior. The use of social media has been observed to be a significant enabler, presenting a favorable image of tourism development; it could thus be used to shape the positive image of tourism development. The effect of social media on tourism development is thus important for changing the overall attitude of the local population.

## Data Availability Statement

The raw data supporting the conclusions of this article will be made available by the authors, without undue reservation.

## Ethics Statement

The studies involving human participants were reviewed and approved by Zhejiang Yuexiu University, Shaoxing, Zhejiang. Written informed consent for participation was not required for this study in accordance with the national legislation and the institutional requirements.

## Author Contributions

All authors listed have made a substantial, direct, and intellectual contribution to the work, and approved it for publication.

## Conflict of Interest

The authors declare that the research was conducted in the absence of any commercial or financial relationships that could be construed as a potential conflict of interest.

## Publisher’s Note

All claims expressed in this article are solely those of the authors and do not necessarily represent those of their affiliated organizations, or those of the publisher, the editors and the reviewers. Any product that may be evaluated in this article, or claim that may be made by its manufacturer, is not guaranteed or endorsed by the publisher.

## Appendix A

**TABLE A1:** Measurement model.

S.	Construct	Items	Scale	Scale
1	Tourism development behavior	4	1. Willing to promote cultural exchanges, with development of CPEC road transport. 2. Willing to support hotels and restaurants, with development of CPEC road transport. 3. Willing to protect natural environmental resources, with development of CPEC road transport. 4. Willing to promote Gilgit-Baltistan as tourist destination, with development of CPEC road transport.	Likert scale 1-5
2	Social Media usage	3	1. I often use social media to obtain CPEC related information and knowledge. 2 regularly use social media to maintain and strengthen communication related with CPEC development. 3. I frequently use social media to share and exchange CPEC related information.	.Likert scale 1-5
3	Overall Attitude:	3	1. In general, the advantages resulting from CPEC development outweigh the disadvantages for the local residents in the region. 2. Generally, I am open to further CPEC development. 3. Generally, CPEC development improves my community living standards.	Likert scale 1-5
4	Community resource concerns	4	1. The CPEC development is a source of negative competition between my community and neighboring communities. 2. Some people and/or groups in the community receive more of the benefits of the CPEC developments than do others. 3. Power is not equally distributed among groups in my community. 4. The CPEC development overtaxes available community financial resources.	Likert scale 1-5
5	Community development	8	1. I support CPEC as having a vital role in this community. 2. CPEC holds great promise for my community’s future. 3. The my community’s government should do more to promote CPEC development. 4. The CPEC development will continue to (or could) play a major economic role in this community. 5. My community should plan and manage the growth of CPEC development. 6. I favor building new facilities through CPEC development that will attract more foreigners. 7. Additional CPEC development would help this community grow in the right direction. 8. CPEC development can be one of the most important industries for a community.	Likert scale 1-5
6	Community satisfaction	3	1. I am pleased environmental change and development that CPEC has created. 2. I am pleased economic development that CPEC has provides to our region. 3. I am pleased social opportunities and possibilities that CPEC has provides to our region.	Likert scale 1-5
